# “Black Esophagus” or Gurvits Syndrome: A Rare Complication of Diabetic Ketoacidosis

**DOI:** 10.1155/2017/4815752

**Published:** 2017-03-05

**Authors:** Vivek Choksi, Kairavee Dave, Rulz Cantave, Sameer Shaharyar, Jeevan Joseph, Uday Shankar, Steven Kaplan, Hamid Feiz

**Affiliations:** Aventura Hospital & Medical Center, Aventura, FL, USA

## Abstract

Acute esophageal necrosis (AEN) also known as “black esophagus” or necrotizing esophagitis is a rare syndrome characterized by a striking diffuse patchy or circumferential black appearance of the esophageal mucosa that preferentially affects the distal esophagus and terminates at the gastroesophageal junction. Only 88 patients over a span of 40 years have received this diagnosis, and the prevalence of this disease ranges from 0.001 to 0.2% of cases in literature. It more commonly affects men (4 : 1 ratio) in the sixth decade of life. It is associated with a high mortality rate, approaching 32%. We report a case of AEN presenting in the setting of diabetic ketoacidosis (DKA), affecting both the proximal and distal esophagus.

## 1. Introduction 

Acute esophageal necrosis (AEN) is commonly referred to as black esophagus or necrotizing esophagitis. The terminology “black esophagus” originates from the typical endoscopic manifestation of AEN [1]. Black necrosis of esophageal mucosa mainly in the distal part of esophagus is observed with histological samples confirming necrotic lesions of mucosa and submucosa. Gastrointestinal bleeding is the most frequent clinical manifestation of AEN. It is often preceded by hemodynamic instability and symptoms of gastroesophageal reflux. Only 88 patients over a span of 40 years have received this diagnosis [2]. A retrospective endoscopy case series demonstrated that the prevalence of acute esophageal necrosis ranges from 0.001 to 0.2% [3]. Males are four times more likely to be affected than females and usually develop in the sixth decade of life [4]. Overall mortality of AEN approaches 32% [2].

## 2. Case Report 

A 65-year-old male with a past medical history significant for coronary artery disease, diabetes mellitus, and peripheral vascular disease presented to the hospital with acute onset of altered mental status, agitation, and confusion. His vitals on admission were temperature 97.8 F, blood pressure 114/55 mm hg, heart rate 84 beats/min, respiratory rate 16/min, and oxygen saturation (SpO_2_) 97% at room air. On physical examination, he was agitated, uncooperative, and alert but not oriented to time, place, and person. His laboratory findings showed blood glucose of 989 mg/dL and arterial blood gas (ABG) showed pH of 7.2, PCO_2_ of 22.2 mmHg, HCO_3_ of 9.8 mEq/L, and PO_2_ of 90.7 mmHg with anion gap of 30, positive serum acetones, urinary ketones of 15 mg/dL, and urinary glucose of >1000. These findings were consistent with the diagnosis of DKA. Imaging studies including CT (head, abdomen, and pelvis) and chest X-ray (CXR) were negative. He was started on insulin drip and supportive care.

**Figure 1:**
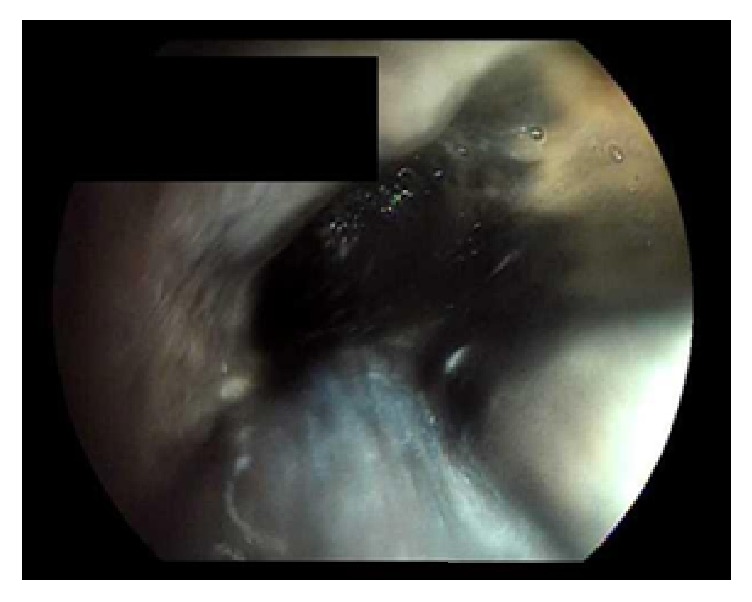
Endoscopic image of proximal esophagus showing circumferentially black mucosa.

**Figure 2:**
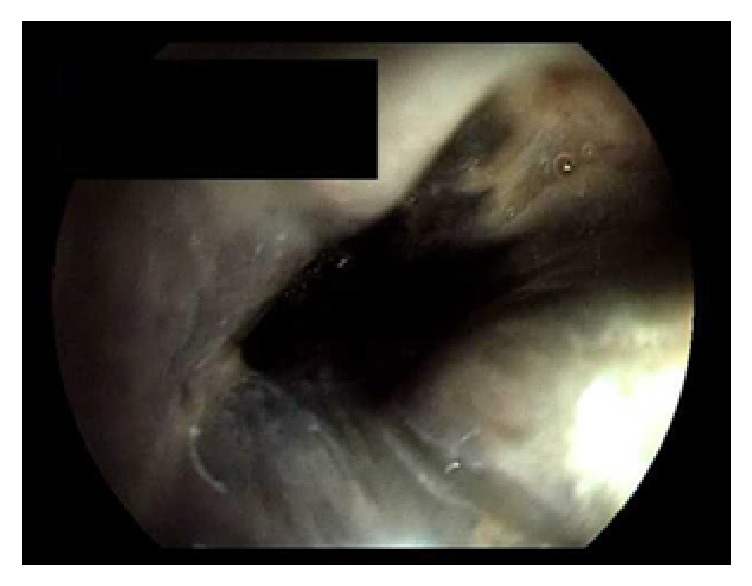
Endoscopic image of proximal esophagus showing circumferentially black mucosa.

On day 3, he developed coffee ground emesis, with a positive fecal occult blood test (FOBT) and melena. His hemoglobin dropped from 10.5 to 7.9 gm/dl. The gastroenterology (GI) service was consulted and emergent esophagogastroduodenoscopy (EGD) was done which demonstrated black, circumferential necrosis of the mucosa of the cervical esophagus without bleeding (Figures 1 and 2). The scope was immediately withdrawn. Endoscopic biopsies were deferred. The patient was kept nil-per-os (NPO) and started on intravenous (IV) fluids, IV proton pump inhibitor (PPI) therapy, IV antibiotics, and IV steroids. Nasogastric (NG) tube insertion was deferred due to the fear of perforation.

**Figure 3:**
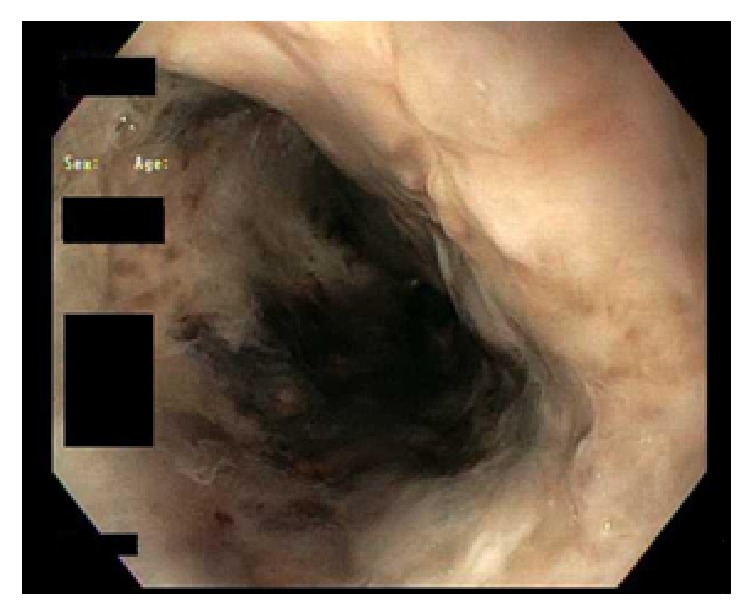
Follow-up endoscopic image (3 days after) of proximal esophagus showing clearing of black color.

**Figure 4:**
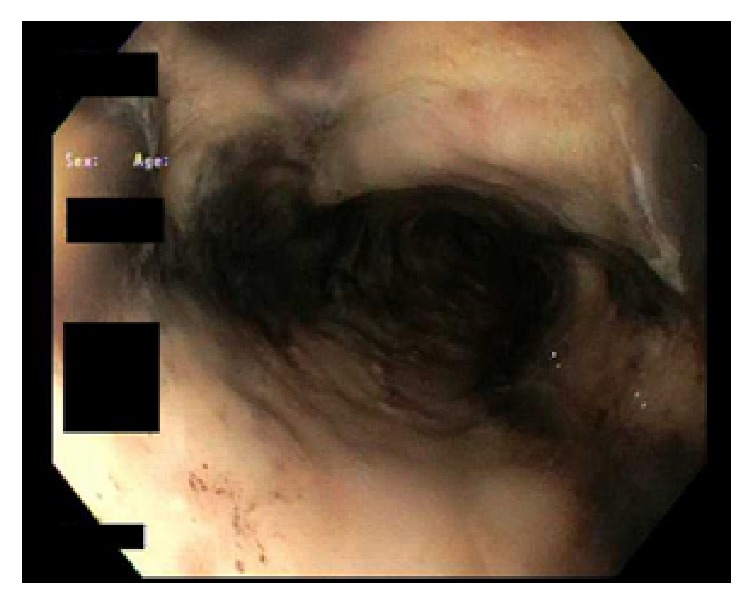
Follow-up endoscopic image (3 days after) of distal esophagus showing esophagitis.

With medical management, the anion gap closed, DKA resolved, and patient was weaned off the insulin infusion. On day 6, a second EGD showed remarkable improvement in the appearance of esophagus but demonstrated severe acute esophagitis (Figures 3 and 4). The entire esophagus from cricopharynx to gastroesophageal junction was ulcerated and eroded with black patchy areas of residual necrosis without any bleeding, stricture, or stenosis. The hypopharynx, stomach, and duodenum were normal. After improvement in patient's condition, on further detailed retrospective history, no history of caustic ingestion was documented. The patient continued to improve and was discharged on day 10. The patient was then lost to follow-up.

## 3. Discussion 

Acute esophageal necrosis (AEN) is a rare clinical disorder, first described by Goldenberg et al. in 1990 and later classified as a distinct syndrome by Gurvits et al. in 2007 [2, 5]. The etiology of AEN is unclear, but ischemia and gastric outlet obstruction may be the inciting events [6]. The pathophysiology of AEN is likely multifactorial and usually results from a combination of tissue hypoperfusion (low flow vascular state), impaired local defense barriers, and massive influx of gastric contents (acid and pepsin) that acutely overwhelm the already vulnerable esophageal mucosa [2].

AEN has been found to be associated with broad-spectrum antibiotic use and concurrent infections (e.g.,* Candida albicans*, cytomegalovirus, herpes virus, and* Klebsiella pneumoniae*) [6]. Additionally, history of diabetes mellitus (24%), malignancy (20%), hypertension (20%), alcohol abuse (10%), and coronary artery disease (9%) places a patient at risk of developing AEN [4].

AEN has never been recorded as a one symptom disorder. Approximately 70% of patients with AEN present with hematemesis. Other symptoms include dysphagia, epigastric pain, chest pain or symptoms related to their underlying disorder, and signs of sepsis, including tachycardia and hypotension [2]. On EGD, AEN is characterized by patchy or circumferential black discoloration with underlying friable hemorrhagic tissue and by a sharp transition to normal-appearing mucosa at the gastroesophageal junction. As the necrosis resolves, the esophagus may become partially covered with thick, white exudates that can be dislodged easily to reveal pink granulation tissue. These exudates most likely represent sloughed mucosa [6].

Currently there is no specific treatment for AEN. Development of AEN generally carries a poor prognosis and the goal of therapy should be directed at treating the coexisting medical diseases. Initial management consists of intravenous hydration, correcting anemia with packed red blood cell transfusion, NPO, and IV PPI. NG tube insertion is contraindicated due to fear of perforation [6]. A decision regarding antimicrobial, antiviral, and antifungal use should be made on an individual basis, especially in critically ill or septic patients as the antibiotic use itself has reported to cause AEN [6, 7].

Other conditions to consider when black esophagus is encountered are melanosis, pseudomelanosis, melanoma, acanthosis nigricans, coal dust, exogenous dye ingestion, lye ingestion, and pseudomembranous esophagitis [6]. Complications of AEN include acute esophageal perforation (<7%), which requires urgent surgical intervention as well as esophageal strictures (>10%), which can occur long after the episode of AEN [2].

Our case reinforces the findings described by Yasuda et al. and shows that one should think of AEN as a differential diagnosis when a 65-year-old or older male presents with DKA and hematemesis [8]. AEN tends to occur in the distal third of the esophagus (97%) due to its relative hypovascularity as compared to the proximal esophagus [2].

After our extensive literature search, we were able to find only four of black esophagus in the English literature, associated with DKA. All of them involved* mid* and* distal* esophagus only [9–12]. Our case was unique, as the necrosis was seen from the beginning of the cervical esophagus, involving both* proximal* and* distal* esophagus, requiring immediate scope withdrawal.

There are limited causes of black esophagus, the most common being caustic ingestion and ischemia. The patient did not ingest any caustic material per history. Therefore diffuse esophageal ischemia was thought to be most likely culprit. We seek to emphasize the idea that the presence of circumferential esophageal necrosis should immediately lead to withdrawal of the endoscope since superficial versus transmural necrosis cannot be distinguished on visual appearance alone. The rarity of this syndrome, coupled with high risk of perforation and mortality, necessitates that physicians be alert to the possibility of encountering this disorder. In addition to lye ingestion, this is one of the few known indications for immediate scope withdrawal and termination of EGD [13].
